# Molecular Model of Prion Transmission to Humans

**DOI:** 10.3201/eid1512.090194

**Published:** 2009-12

**Authors:** Michael Jones, Darren Wight, Rona Barron, Martin Jeffrey, Jean Manson, Christopher Prowse, James W. Ironside, Mark W. Head

**Affiliations:** University of Edinburgh, Edinburgh, Scotland, UK (M. Jones, D. Wight, R. Barron, J. Manson, J.W. Ironside, M.W. Head), Veterinary Laboratory Agency, Edinburgh (M. Jeffrey); Scottish National Blood Transfusion Service, Edinburgh (C. Prowse)

**Keywords:** Transmissible spongiform encephalopathy, disease transmission, model system, prions, amino acid sequence, conformation, molecular characteristics, protein misfolding cyclic amplification, dispatch

## Abstract

To assess interspecies barriers to transmission of transmissible spongiform encephalopathies, we investigated the ability of disease-associated prion proteins (PrP^d^) to initiate conversion of the human normal cellular form of prion protein of the 3 major *PRNP* polymorphic variants in vitro. Protein misfolding cyclic amplification showed that conformation of PrP^d^ partly determines host susceptibility.

The agents responsible for the transmissible spongiform encephalopathies (TSEs) are called prions. Although their precise biochemical composition is a matter of debate, they are known to occur in a series of strains, each with a characteristic disease phenotype and host range (*1*). A central event in neuropathogenesis of TSEs is conversion of the normal cellular form of the prion protein (PrP^C^) to the pathognomonic disease-associated isoform (PrP^d^) (*2*). In the absence of a known nucleic acid genome, it has been proposed that the strain-like properties of different TSE agents are encoded by distinct self-propagating conformational variants (conformers) of PrP^d^ (*3*). The best developed method available for typing these PrP^d^ isoforms uses limited proteolysis and classification of the protease-resistant prion protein (PrP^res^) in terms of the sizes of the nonglycosylated fragment(s) produced and the ratio of the 3 possible glycoforms (*3*). If distinct conformers and glycotypes of PrP^d^ are responsible for diversity of prion strains, then they would be expected to be able to impose these molecular characteristics onto PrP^C^ of the same amino acid sequence (when transmitted or replicating within a species) and onto PrP^C^ of a different primary sequence (when transmitted between species). In support of this theory, the agent responsible for the TSE of cattle, called bovine spongiform encephalopathy (BSE), the accepted cause of variant Creutzfeldt-Jakob disease (vCJD) in humans (*4*), has been shown to be transmissible to at least 7 species (*1*), resulting in propagation of PrP^d^ that retains the characteristic molecular signature of the original BSE prion strain (*5*–*7*).

Current thinking favors a seeded polymerization model for the conversion of PrP^C^ into PrP^d^, which has led to the development of several cell-free in vitro conversion model systems (*8*). One such system is protein misfolding cyclic amplification (PMCA) (*9*), in which small amounts of PrP^d^ introduced (seeded) into substrate containing excess PrP^C^ and other essential conversion cofactors can be amplified to readily detectable levels by sequential cycles of sonication and incubation. We have previously reported that the molecular characteristics, electrophoretic mobility, and glycoform ratio of the PrP^res^ associated with the vCJD PrP^d^ conformer were faithfully reproduced by PMCA (*10*). However, the efficiency of amplification achieved depended on the substrate’s prion protein gene codon 129 (*PRNP*-129) genotype. The most efficient amplification was achieved in a methionine homozygous (*PRNP*-129MM) substrate; the least efficient, in a valine homozygous (*PRNP*-129VV) substrate. To estimate the molecular component of transmission barriers for particular TSE agents between species, we used PMCA reactions to amplify PrP^d^ associated with vCJD (*10*), bovine BSE (*11*), ovine scrapie (*12*), and experimental ovine BSE (*13*) and substrates prepared from humanized transgenic mouse brain tissue expressing each of the 3 main *PRNP* polymorphic variants found in Caucasian human populations (*PRNP*-129MM, MV, and VV) (*14*).

## The Study

We prepared seed and substrate homogenates as 10% (wt/vol) homogenates in PMCA conversion buffer (*10*). Seed homogenates were diluted into substrate homogenates so that all PMCA reactions contained equivalent amounts of PrP^d^ based on the PrP^res^ levels in each seed homogenate. PrP^res^ levels were determined by Western blot titration that used monoclonal antibody (MAb) 6H4 after limited proteinase K digestion. The reaction mixes were split into 2 aliquots; 1 aliquot was stored immediately at –80°C (−PMCA), and the other was subjected to 48 cycles of PMCA (+PMCA) (*10*). To assess the degree of PrP^d^ amplification achieved from each seed in each substrate, the samples −/+ PMCA were subjected to limited proteinase K digestion, and PrP^res^ was detected by Western blotting with MAb 6H4 (which recognizes human, bovine, and ovine PrP) and MAb 3F4 (which selectively recognizes only human PrP and would therefore specifically identify PrP^res^ formed from human PrP^C^).

**Figure 1:**
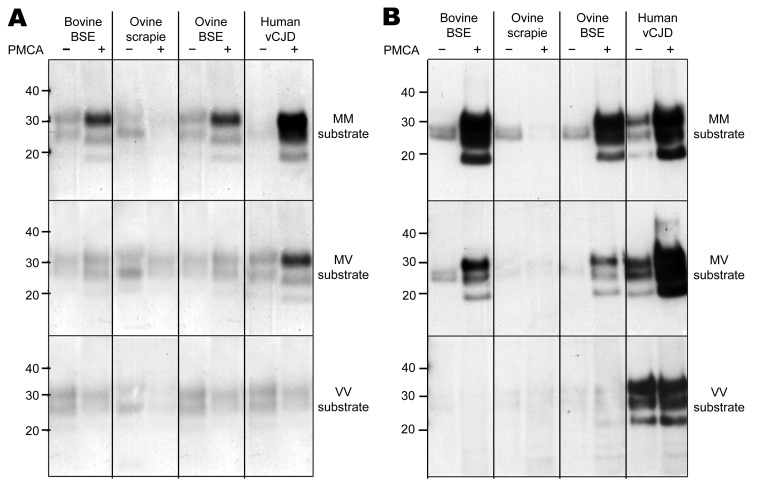
Amplification of PrP^d^ by PMCA from bovine BSE, ovine scrapie, experimental ovine BSE, and human vCJD brain homogenates in substrate homogenates prepared from humanized transgenic mouse brain tissue expressing PrP of each human prion protein gene codon 129 (*PRNP*-129) genotype. A) Amplification of each PrP^d^ type, as determined by Western blotting using MAb 6H4 to detect PrP^res^ after limited proteinase K digestion, in a *PRNP*-129MM substrate (top panel, 3-min exposure), a *PRNP*-129MV substrate (middle panel, 3-min exposure), and a *PRNP*-129VV substrate (bottom panel, 3-min exposure). B) Amplification of each PrP^d^ type, as determined by Western blotting using MAb 3F4 to detect PrP^res^ derived from human PrP after limited proteinase K digestion, in a *PRNP*-129MM substrate (top panel, 30-s exposure), a *PRNP*-129MV substrate (middle panel, 3-min exposure), and a *PRNP*-129VV substrate (bottom panel, 10-min exposure). Limited proteinase K digestion and Western blotting were conducted out as previously described (*11*). MAb 6H4 (Prionics, Schlieren-Zurich, Switzerland) and MAb 3F4 (Dako, Ely, Cambridgeshire, UK) were used at a final concentration of 50 ng/mL. PrP^d^, disease-associated prion protein; PMCA, protein misfolding cyclic amplification; BSE, bovine spongiform encephalopathy; vCJD, variant Creutzfeldt-Jakob disease; MAb, monoclonal antibody; PrP^res^, protease-resistant prion protein; MM, methionine homozygous; MV, methionine/valine heterozygous; VV, valine homozygous. Values on the left are in kilodaltons.

**Figure 2:**
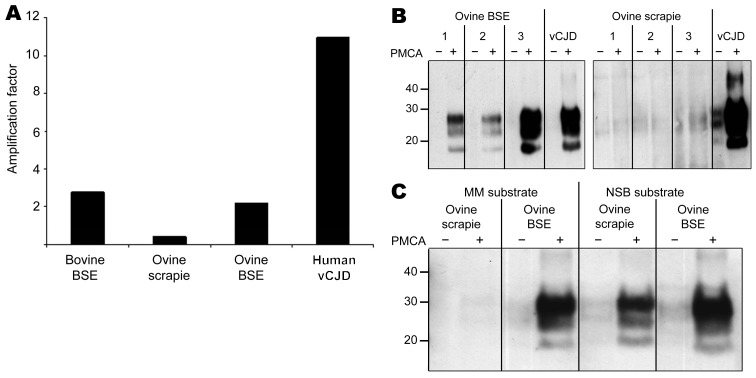
A) Semiquantitative densitometric analysis (optical density × area in mm^2^) of Western blot data (Figure 1, panel A, top panel), showing the amplification factors (+PMCA/−PMCA) obtained for all 4 seeds (bovine BSE, ovine scrapie, ovine BSE, and human vCJD in the *PRNP*-129MM substrate. B) Amplification of PrP^d^ associated with ovine BSE (left) and ovine scrapie (right) from each of 3 different sheep in *PRNP*-129MM substrate as determined by Western blotting using MAb 3F4 to detect PrP^res^ after limited proteinase K digestion. Substrate was seeded with brain homogenates prepared from sheep with confirmed scrapie and BSE such that each PMCA reaction mix contained an equivalent amount of PrP^d^ according to detection of PrP^res^ by Western blot titration after limited proteinase K digestion. *PRNP*-129MM substrate seeded with vCJD brain homogenate was included as a positive control in each experiment. C) Amplification of PrP^d^ associated with ovine scrapie and BSE in substrates prepared from *PRNP*-129 methionine homozygous humanized transgenic mouse brain tissue (MM substrate) and NSB substrate. Substrates were prepared as 10% (wt/vol) homogenates in PMCA conversion buffer (*10*). Each substrate was seeded with brain homogenates prepared from sheep with confirmed scrapie and BSE so that each PMCA reaction mix contained an equivalent amount of PrP^d^ as determined by detection of PrP^res^ by Western blot titration after limited proteinase K digestion. Reaction mixes were divided into 2 lots: 1 was stored immediately at –80°C (−PMCA) and the other was subjected to 48 cycles of PMCA (+PMCA) by using standard conditions (*10*). After limited proteinase K digestion, PrP^res^ in samples −/+PMCA was detected by Western blotting using MAb 6H4. PMCA, protein misfolding cyclic amplification; BSE, bovine spongiform encephalopathy; vCJD, variant Creutzfeldt-Jakob disease; MM, methionine homozygous; PrP^d^, disease-associated prion protein; MAb, monoclonal antibody; PrP^res^, protease-resistant prion protein; NSB, normal ARQ/ARQ sheep brain tissue. Values on the left in panels B and C are in kilodaltons.

Using MAb 6H4 to probe Western blots, we noted amplification of vCJD, bovine BSE, and ovine BSE PrP^res^ in the *PRNP*-129MM substrate (Figure 1, panel A, top) but not in the *PRNP*-129VV substrate (Figure 1, panel A, bottom). Semiquantitative assessment of these Western blots by densitometry showed that the degree of amplification of vCJD PrP^res^ was considerably greater than that of bovine or ovine BSE in the *PRNP*-129MM substrate (Figure 2, panel A). A more sensitive and discriminatory Western blot conducted by using MAb 3F4 confirmed efficient amplification of vCJD, bovine BSE, and ovine BSE PrP^res^ in the *PRNP*-129MM substrate (Figure 1, panel B, top), weaker amplification in the *PRNP*-129MV substrate (Figure 1, panel B, middle), and little, if any, amplification in the *PRNP*-129VV substrate (Figure 1, panel B, bottom). In all substrates, the amplified PrP^res^ retained the electrophoretic mobility and glycoform ratio associated with BSE-related PrP^res^. No amplification of ovine scrapie PrP^res^ was evident after PMCA in any of the *PRNP* humanized transgenic mouse brain substrates (Figure 1, panels A, B). The difference between ovine scrapie and ovine BSE in ability to seed amplification in *PRNP*-129MM substrate was a robust phenomenon evident in brain samples from 3 different ARQ/ARQ sheep with each disease (Figure 2, panel B). However, failure of the ovine scrapie seed to amplify was not caused by a general lack of competence to do so or by inappropriate amplification conditions because robust amplification of ovine scrapie PrP^res^ was evident after PMCA in a substrate prepared from normal ARQ/ARQ sheep brain (Figure 2, panel C).

## Conclusions

Our results are best appreciated in terms of the molecular interaction between seed PrP^d^ and substrate PrP^C^, specifically the species-specific amino acid sequence and *PRNP* polymorphic status of PrP^C^ and PrP^d^ and the PrP^d^ conformers involved (Table). Regardless of the seed PrP amino acid sequence, the PrP^d^ conformers associated with bovine BSE, ovine BSE, and human vCJD were amplified in the humanized mouse substrate and displayed similar *PRNP*-129 genotype preferences (*PRNP*-129MM >*PRNP*-129MV >*PRNP*-129VV). In contrast, the PrP^d^ conformer associated with the ovine scrapie strain, although sharing the same PrP amino acid sequence as the PrP^d^ in ovine BSE, could not be amplified in any of the *PRNP* humanized mouse substrates but could be amplified in a sheep brain substrate. These observations are consistent with conformation of a TSE agent’s PrP^d^ (rather than solely its amino acid sequence) having a role in determining the susceptibility of a host’s PrP^C^ to conversion. They similarly suggest that these molecular factors could in turn have a powerful influence on disease susceptibility and incubation time.

**Table:** Summary of the properties of the sources used in PMCA of vCJD, bovine BSE, ovine scrapie, and experimental ovine BSE PrP^res^*

Seed homogenate	Species	Bovine†	Human‡	Ovine§	Ovine§
	Disease	BSE	vCJD	BSE	Scrapie
	Tissue	Brain	Brain	Brain	Brain
	*PRNP* amino acid sequence	Bovine	Human	Ovine	Ovine
	*PRNP* polymorphism	140MM	129MM	ARQ/ARQ (132MM)	ARQ/ARQ (132MM)
	PrP^d^ “conformer”	BSE	BSE	BSE	Scrapie
Substrate homogenate¶	Species	Mouse	Mouse	Mouse	Mouse
	Tissue	Brain	Brain	Brain	Brain
	PrP amino acid sequence	Human	Human	Human	Human
	*PRNP*-129 polymorphism	MM, MV, and VV	MM, MV, and VV	MM, MV, and VV	MM, MV, and VV
	Background genotype	129 Ola *prnp*^−/−^	129 Ola *prnp*^−/−^	129 Ola *prnp*^−/−^	129 Ola *prnp*^−/−^

*PMCA, protein misfolding cyclic amplification; vCJD, variant Creutzfeldt-Jakob disease; BSE, bovine spongiform encephalopathy; PrP^res^, protease-resistant prion protein; PrP^d^, disease-associated prion protein; MM, methionine homozygous; MV, methionine/valine heterozygous; VV, valine homozygous. †Bovine brain tissue was sampled from brain tissue taken from a Friesian cow with terminal BSE (*11*). ‡Human brain tissue (frontal cortex) was sampled from a frozen half brain that had been collected at autopsy with the appropriate consent for tissue retention and research use from a patient methionine homozygous at *PRNP* codon 129, who received a final diagnosis of definite vCJD by established criteria. Ethical approval for its use in this study was covered by LREC 2000/4/157 (J.W.I.). §Both the ovine scrapie (*12*) and ovine BSE (*13*) brain tissue (hind brain) were sampled from clinically sick sheep. The distinctive disease phenotypes were confirmed by histopathologic, immunohistochemical, and Western blot characteristics. ¶Frozen half brains from inbred transgenic mouse lines expressing human PrP of the 3 major *PRNP* codon-129 genotypes (MM, MV, VV) were used to prepare substrate homogenates. These mice had identical genetic backgrounds, were produced to express human PrP by direct replacement of the murine PrP gene, and all expressed equivalent amounts of human PrP regardless of the *PRNP*-129 genotype (*14*). The transgenic mice were bred under license to the UK Home Office in accordance with the UK Animals (Scientific Procedures) Act of 1986, and the use of brain tissue from these mice was reviewed and approved by the local Ethics Review Committee.

To date, all clinical cases of vCJD have occurred in persons with the *PRNP*-129MM genotype, as might be predicted from the efficiency of amplification of BSE-related PrP^d^ shown here. Extrapolating from these results, one would predict that the next genotypic group most likely to show susceptibility to the BSE agent would be heterozygous (MV) at codon 129 of the *PRNP* gene, as previously suggested from the corresponding in vivo transmission studies (*14*).

In the wake of BSE epidemics in the United Kingdom and elsewhere, enhanced surveillance has identified apparently new TSEs (*15*), raising concerns regarding animal and human health. PMCA with suitable substrate sources could provide a rapid way to estimate the molecular component of transmission barriers for particular TSE agents between species, including humans. These estimates could thus indicate whether, like classical scrapie, the agents represent little risk for human health or whether, like classical BSE, they represent cause for concern.
